# An Account of the Yellow Fever Which Appeared in the City of Galveston, Republic of Texas, in the Autumn of 1839, with Cases and Dissections

**Published:** 1841-10

**Authors:** 


### Art. IX.

*An Account of the Yellow Fever which appeared in the City of Galveston, Republic of Texas, in the Autumn of* 1839, *with Cases and Dissections.* By Ashbel Smith, m.d. a.m., Ex-Surgeon-General of the Texian Army.—*Galveston, Texas,* 1839. 8vo, pp. 78.

The intrinsic merit of this little unpretending volume, and the opportunity which it affords us of comparing together yellow fever as it exists in points so remote from each other as Gibraltar* and Texas, invest it with a considerable degree of interest; but a portion of the feeling with which we have contemplated it, is certainly derived from the circumstances of the distant region whence it has been transmitted to us, the

* See our Review of M. Louis' work on the same subject in our Twentieth Number.

young republic of Texas. The possession of this district by its present occupants and rulers was described by Walter Savage Landor, as "the most notable theft of modern times." Dr. Smith's book shows, however, that this theft has that redeeming quality about it with which political thefts alone are sometimes accompanied, that of being the means of diffusing civilization—through the great instruments of such diffusion, the Anglo-Saxon race—to regions which had not previously enjoyed this blessing.

The city of Galveston, the scene of the epidemic, though scarcely two years old, contains above 2,000 inhabitants. It is in lat. 29° 18′ n., and long. 96° 6′ w. of Greenwich. The town extends quite across the island of Galveston on which it is situated, and which at this point varies from a mile and a quarter to two miles. The city is thus washed on its south-eastern border by the gulf of Mexico, whilst its north-western side is washed by Galveston Bay, a broad sheet of water. The harbour is on the side of the island fronting this bay. The heaving of the tide has formed a natural mole (*levée*) along the shore of the harbour of about two feet in height and one hundred in breath, and behind this levée the land is low, being nearly on a level with the water at middle tide, and overflowed in high tides. From this overflowing of the tides and occasional rains there exists at all times either a quagmire or a sheet of shallow water three fourths of a mile long, and varying from one hundred to three hundred feet in breadth, exposed to the rays of an ardent sun, between the mole and the higher land of the island. Immediately contiguous to this morass is the Strand, the principal business street of the town, in which street the disease was first observed.

Of the general salubrity of the island, Dr. Smith speaks in warm terms, regarding it, in this respect, as unsurpassed by any place in the world. It is exempt, he informs us, from the typhus fever of cold climates, and the malignant endemics of the miasmatic regions of the south. The few diseases that occur there are for the most part of a moderately inflammatory character, and readily yield to the simplest treatment. The range of the thermometer is high during the warm season, but the bland breezes from the south which prevail very commonly throughout this period, and are usually strongest at midday, generally prevent the heat from being oppressive.

Between Dr. Smith's description of the yellow fever of Galveston, and that furnished by M. Louis, of the disease in Gibraltar, in 1828, there is the closest resemblance. There were first pains in the limbs, some sickness at stomach, chills not amounting to complete rigor, and some diminution of the sensibility of the extremities. In a variable period ranging from two or three minutes to a few hours, there succeeded pain in the forehead and eyes, pains in the loins, great restlessness, blood-shot eyes, flushed face, hot and dry skin, and full and frequent pulse; whilst the pains in the limbs and sickness of the stomach which were present at first still continued. The tongue was moist and sometimes furred and swollen, but not unfrequently of a healthy aspect. The thirst was often moderate, in some cases considerable, never intense. The epigastrium was slightly sensible on pressure in some cases, in others quite free from pain. The mental operations were generally coherent, but sluggish. There was this to be remarked in the restlessness, that it did not consist in jactitation, but in a disposition to rise from bed and walk about.

A diminution of pain and febrile excitement very generally takes place, from eight or ten to twenty-four hours after the invasion. If the disease proceed to a favorable termination, this abatement is progressive, and convalescence takes place at a period varying from the third to the fifth or seventh day, or as was observed in one case, to the fifteenth day. Cases terminating thus favorably and speedily correspond as closely as possible with the mild cases observed by Louis in the epidemic of Gibraltar. The symptoms in fatal cases are graphically described, and they will be found to bear to those observed at Gibraltar a resemblance which places the identity of the diseases beyond all question. We regret that we cannot afford space to lay the description before our readers. The author considers pains in the head, eyes, and loins; the characteristic expression of the eyes; vascular excitement and gastric irritability in the first periods; augmented gastric irritability and black vomit near the fatal termination, and yellow suffusion after death as the pathognomonic symptoms of the disease. It sounds somewhat odd to hear a cadaveric appearance classed among these pathognomonic symptoms, but it should be remarked that there are various natural diseases besides yellow fever, in which the appearances after death are very characteristic, spasmodic cholera for example.

So far there is a correspondence between the description of the disease at Gibraltar and that at Texas sufficiently complete to prove the identity of the diseases in external character; indeed the discrepancy is no greater than might have occurred between the sketches by different pencils of the same complex object. The same exact correspondence, however, does not exist between the pathological lesions as portrayed by the respective writers; and a summary of what Dr. Smith observed in his anatomical researches will render this manifest; still, as Dr. Smith seems to have examined only *eight* cases, we are disposed to think the discrepancy between the two observers would have been lessened had the investigations been more extensive.

Other structures, Dr. Smith remarks, experience the fury of the disease, and contribute to the mortality, but the mucous coat of the stomach is the tissue on which the disease *uniformly* and mainly commits its greatest ravages. On pouring off the black vomit, which the stomach in all fatal cases contained, and detaching from the mucous coat the adherent dark-coloured *flocculi*, this tissue was found of a dull pearlish white colour, thickened and softened. In some cases the softening was so great that the villous coat could be scraped in portions almost into a pulp with the finger. The thickening was not uniform but presented in portions *rugæ*, and an uneven surface somewhat like the "unevennesses" of the rind of a lemon. These irregularities in the mucous coat doubtless correspond with the mamellonated appearance described by Louis at Gibraltar. There were a few points and some scattered stelliform particles of bright red; but these points and patches would not, except in a single case, form by their aggregation a surface of an inch square. In two of the cases examined, the whole mucous coat of the stomach presented the white, much-thickened, and softened condition above described; but in four cases, from three fourths to five sixths only of the mucous coat presented this condition, commencing at the pylorus, and terminating within one or two inches of the cardiac orifice, whilst the remaining portion surrounding the cardia was the seat of a most intense, diffuse, red injection, preserved its usual firmness, was but little if at all thickened, and entirely destitute of flocculi adherent to the surface. This injection did not present pointed or stellated patches, but the blood appeared to be diffused throughout the mucous tissue, and the colour was more or less intense in proportion to the quantity of blood contained in the different parts, and the hue was that between venous and arterial blood. The line of demarcation between the pale or colourless and injected portions of the mucous coat was, for the most part, as well defined by the different thickness of the two portions as by their different colour; the white portion being thickened, whilst the red and engorged part still preserved its normal thickness.

The author's description of the condition of the intestines, or, more properly speaking, of their contents, corresponds very closely with that of Louis, excepting that at Texas the glands of Brunner and Peyer were more frequently and considerably affected than they appear to have been at Gibraltar, for Dr. Smith informs us that they were " sometimes greatly developed," whilst from Louis we learn that in one case only were the patches of Peyer near the *cæcum* slightly tumefied, and with this exception there was no affection of the glands of Brunner and Peyer.

The liver was found in all cases, says Dr. Smith, of its usual dimensions of ordinary firmness, and without any obvious structural derangement. *In three cases it was of a very light drab colour, externally and internally*, and destitute of blood, in one of a dark claret colour and congested with blood, and in the others of its usual appearance, and containing a moderate quantity of blood. In all cases there appeared to be a suspension of the biliary secretion; no bile could be squeezed from the substance of the liver. The author quotes a case, but, as he admits needlessly, to contradict the popular error that the black vomit is a vitiated secretion of this organ. In this case the *ductus communis* contained a little yellow bile, while the stomach was full of turbid black vomit of the deepest dye.

The author's conclusion from his pathological researches is, that two important organs have invariably suffered, the stomach and liver. The mucous coat of the former organ always has presented severe structural derangements, a condition which he regards as the proximate cause of the death of the individual, the *mortis ratio sufficiens;* whilst the latter organ, the liver, has with equal uniformity exhibited undoubted evidences of severe functional derangment, a total suspension of biliary secretion. The difference between his researches into the condition of the stomach, and conclusions from them, and those of Louis, consists in this, that the latter regards " the different lesions of the gastric mucous membrane as secondary or accessory, and that *in cases where they are found,* they were probably developed at a certain period after the commencement of the disease;"* and that whilst Dr. Smith considers these lesions indicated by thickening and softening of the tissue as universal, M. Louis says expressly, that the thickness of the membrane was natural in half the cases;

* Louis on the Yellow Fever of Gibraltar, translated by Dr. Shattuck, (p. 99.)

in others it was thickened; and that its consistence was normal in thirteen cases, in ten it was softened. Then with regard to the liver, though Dr. Smith considers its functional derangement, suspension of biliary secretion, as uniformly existing, he mentions expressly that the anemic condition and light colour of this organ were found in three cases only out of seven: but this condition and colour M. Louis found invariably existing, and finding the liver thus constantly affected, he pronounces it to be the anatomical character pathognomonic of the disease. We must suppose this difference to exist in the things observed not in the observers. Our opinion of Louis has been too often expressed to require repetition here; and Dr. Smith's book contains ample internal evidence that he too is an accurate observer and a faithful reporter.

In the section on Treatment, the author gives a statement, very creditable for its candour, of the difficulties he experienced on the first occurrence of the epidemic; his trial of the remedies, calomel especially, which he had found successful in the more ordinary diseases of the climate; their failure, his disappointment, and difficulty in attaining to a more successful practice. The inspection of three bodies, he tells us, disclosed to him the awful ravages of the disease on the stomach; mercurials had failed to procure stools, and he sought a medicine which should unload the alimentary canal without irritating the gastric membrane: this he finds in an infusion of senna and rhubarb, in which manna is dissolved. His treatment consists in bloodletting employed as soon as the excitement is fairly developed, till slight faintness is produced; hot and strong mustard-baths applied to the feet and legs; the patient is placed in bed, and most sedulously guarded against any current of cold air, which may repel the blood from the surface; and the cathartic already mentioned is administered till its operation is produced. After this no other medicine is required in the stage of excitement, but the hot mustard-bath is repeated twice or three times in the twenty-four hours, as a means of sustaining a continued glow on the surface, whilst a little tamarind-water or sage-tea is given as a beverage.

If after the abatement of excitement the extremities become cool, the hot mustard-bath should be promptly resorted to, and nausea and vomiting occurring should be allayed with black-drop or laudanum. Opiates in this advanced stage are very useful, and have appeared to save some who were rapidly sinking. The author's great reliance, however, is upon bleeding employed early, the mild cathartic, mustard-baths, mild beverages, and a room of moderate temperature and properly ventilated, the patient being at the same time secured from the direct impression of currents of air which may repel the blood from the surface. Under this seemingly judicious treatment, a great majority of Dr. Smith's patients recovered; but as he gives neither the actual number treated nor the ratio of recoveries, we are unable to pronounce whether it was very successful or not.

The question of contagion is treated briefly but forcibly. Yellow fever had been prevailing for some time at New Orleans when it appeared at Galveston, and there is much intercourse between the places. On the other hand, what Dr. Smith considers the local causes of the disease were very abundant about the limited district, the Strand, to which the infection was confined. There were animal and vegetable matters abounding around the houses, and a marsh exposed to the heat of the sun, when the temperature ranged daily in the shade from 84° to 89°. Patients were removed from the infected district to the healthy sections of the city without communicating the disease to their attendants, or the inmates in their new abode; on the contrary, these sections retained throughout the epidemic, their unsurpassed healthfulness. Of the medical men of the place two only were attacked, and they alone dwelt in the infected district. We consider these points as very important. Dr. Smith performed many dissections, examining everything closely, and immersing his hands freely in black vomit, &c., but with perfect impunity. He *tasted* black vomit repeatedly when fresh ejected from the stomach of the living with equal impunity.

The conclusion the author arrives at seems to be perfectly justified by these premises:

"After a careful observation of the history of the epidemic, no fact has come to light which would show that the disease was contagious, that is, communicable from a person labouring under it to one in health; but that it is contracted only by exposure in the infected district." (p. 33.)

To conclude, we beg to say that this first brief but vigorous specimen of Texian literature has pleased us exceedingly; and that we shall be gratified to observe the future achievements of Dr. Smith and other sons of the young republic in the walks of medical learning, fulfilling the promise of these first fruits.

---

